# The Effect of the Covid-19 Pandemic on Global Armed Conflict: Early Evidence

**DOI:** 10.1177/1478929920940648

**Published:** 2021-05

**Authors:** Marius Mehrl, Paul W Thurner

**Affiliations:** 1 Department of Government, University of Essex, Colchester, UK; 2 Geschwister-Scholl-Institut für Politikwissenschaft, Ludwig-Maximilians-Universität München, Munich, Germany

**Keywords:** Covid-19, armed conflict, coronavirus, Civil War

## Abstract

As Covid-19 spreads around the world, international actors, including the United Nations, have called for a stop to armed conflict to facilitate efforts to fight the pandemic. At the same time, coronavirus may also trigger and intensify armed conflict due to its negative economic consequences and by offering windows of opportunity to opposition movements to attack distracted and weakened incumbents. We use real-time data on the spread of Covid-19, governmental lockdown policies, and battle events to study the causal short-term effect of the pandemic on armed conflict. Our results suggest that both the spread of Covid-19 and lockdown policies exhibit a global Null effect with considerable regional heterogeneity. Most importantly, governmental lockdowns have increased armed conflict in the Middle East. In contrast, reported combat has decreased in Southeast Asia and the Caucasus as the pandemic has spread.

## Introduction

On 23 March 2020, the Secretary-General of the United Nations, António Guterres, called for a global ceasefire to ‘create corridors for life-saving aid[,] open precious windows for diplomacy’, and thus facilitate stopping the spread of Covid-19 among vulnerable populations in war-torn countries (cited in UN, 2020). Continued armed conflict would hinder efforts to fight coronavirus and thus act as a catalyser. At the same time, the pandemic may trigger and fuel fighting due to its negative economic consequences and the windows of opportunity it offers to opposition movements. We study the short-term effect of Covid-19 on armed conflict within a difference-in-difference framework, leveraging temporarily fine-grained data on the spread of Covid-19, governmental responses, and battle events. Our results indicate a global null effect of the pandemic on armed conflict; while fighting in the Caucasus and Southeast Asia has decreased in the wake of the first reported cases, governmental lockdowns have intensified conflict in the Middle East.

## Background

Existing studies document substantial and long-lasting effects of armed conflict on public health outcomes, including the prevalence of infectious diseases (Bundervoet et al., 2009; Ghobarah et al., 2003; Hendrix and Gleditsch, 2012). Continued armed conflict thus has the potential to fuel the spread of Covid-19 and be a key barrier to halting it. This is why the United Nations have been emphasizing the need to cede fighting, and it makes the announcement of ceasefires in, for example, the Philippines, Libya and Colombia, a reason for optimism. However, the number of such ceasefires has remained limited and some of them were broken shortly after being announced (Rustad et al., 2020). At the same time, some analysts argue that Covid-19 may lead to a ‘Pax Epidemica’ even without ceasefires as it decreases states’ military capabilities and optimism to fight (Posen, 2020). While such a decrease in fighting would clearly facilitate efforts to tackle the pandemic, it remains unclear whether a reduction in violence is actually occurring.

Instead, it is also possible that the virus is fuelling armed conflict in currently unrecognized ways. The global economy is already experiencing substantial contractions as a result of Covid-19. With most commodity prices dropping (World Bank, 2020), developing countries are expected to be particularly affected and to see an increase in poverty (Melaine and Nonvide, 2020; Noy et al., 2020). Numerous studies suggest that worsened economic conditions can trigger and intensify fighting as economically deprived individuals are recruited into rebel groups (Brückner and Ciccone, 2010; Chaudoin et al., 2017; Collier and Hoeffler, 2004; Humphreys and Weinstein, 2008). The pandemic may therefore indirectly increase armed conflict due to its effects on the economy. For instance, violent protests have already erupted in Lebanon over the economic consequences of Covid-19 and of the government-imposed shutdown to stop it (*ABC News*, 2020), while in Yemen and Somalia, rebel groups are seeking to recruit fighters among the deprived (Blanc, 2020; Nagi, 2020).

At the same time, opposition groups intending to challenge the state may view coronavirus as a window of opportunity as their target is focused on taking measures against the pandemic. This is especially the case if these measures are perceived to fall short, thus signalling state weakness. In this vein, the Yemeni Southern Transitional Council explicitly pointed to the central government’s failure to prepare for an outbreak of the virus when announcing its breakaway and self-administration of the territory it holds (Erhardt, 2020). In addition, the pandemic has resulted in external intervenors in the conflicts in Syria and Iraq curtailing operations or even pulling out their troops entirely (Hasan, 2020; Yahya, 2020), thus opening up the field to increased rebel activity. States’ reduced ability to fight and project power may thus not necessarily lead to peace (cf. Posen, 2020), but instead help their non-state challengers (Bagozzi, 2016).

In the following, we thus examine whether the Covid-19 outbreak has led to a reduction of armed conflict, as called for by the UN Secretary-General, or instead fuelled it.

## Research Design

**Figure 1.**
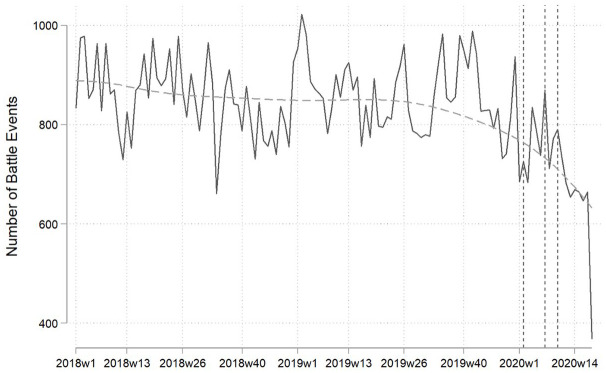
Global Battle Events, January 2018–April 2020. Solid and dashed horizontal lines present the actual and smoothed weekly number of battles. Smoothing uses locally weighted scatterplot smoothing (bandwidth: 0.8). Dashed vertical lines indicate weeks 2020w2, 2020w7 and 2020w10 where the first, 1000th, and 10,000th case of coronavirus were reported.

To study this question, we rely on real-time data on battle events and the spread of Covid-19 from the Armed Conflict Location and Event Dataset (ACLED, Raleigh et al., 2010) and the Oxford Covid-19 Government Response Tracker (Hale et al., 2020). We aggregate observations to the country-week level as reporting quality is likely to differ between weekdays and weekends. Based on these sources, Figure 1 presents the global time-series of battle events covering the period from January 2018 to the last week of April 2020.^1^

The figure suggests that the number of battles decreased during the Covid-19 pandemic, as their weekly numbers are lower in the period after the first case than in that before and have been almost monotonically declining since the 1000th case was reported. The number of battle events during the pandemic is also lower than in the same months in the years 2018 and 2019. Figure 1 thus presents some evidence that global armed conflict, if measured by the reported number of weekly battles, has decreased during the coronavirus pandemic. However, simply comparing battle numbers across years can only be a start as a variety of factors, not only the incidence of Covid-19, may differ between the years. For instance, countries at conflict in 2020 may have been peaceful in 2018–2019 and vice versa. Similarly, the number of total active conflicts will most likely not be constant across all 3 years.

Next, we thus examine the effect of Covid-19 on armed conflict more formally by leveraging differences in when countries were affected by – and responded to – coronavirus within a difference-in-difference framework (DiD). This modelling strategy allows us to estimate the causal effect of Covid-19 on armed conflict while purging the effects of a number of confounders from the analysis. While the canonical DiD compares observations from two units, one treated and one control, across two time-periods, we employ the generalized version with more than two periods and multiple units which vary in treatment timing (Angrist and Pischke, 2009). In other words, identification relies not on there being one or more countries which are never affected by coronavirus but instead exploits the fact that the pandemic spread to different countries at different points in time. This set-up is represented by the following equation: $\mathrm{battle}\;\;\mathrm{even}t_{iw}=\alpha_{i}+\gamma_{w}+\beta T_{iw}+\varepsilon_{iw}$ (Cameron and Trivedi, 2005: 768–769),^2^ where $\mathrm{battle}\;\;\mathrm{even}t_{it}$ is the number of battle events in country i and week w, $\alpha_{i}$ and $\gamma_{w}$ are country- and week-fixed effects, and $T_{iw}$ is a dummy indicating treatment status. We are interested in the coefficient of this binary item, β, and use two different treatments. First, we use a dummy that takes the value one in the week a country reports its first Covid-19 case as this is arguably the clearest signal that the pandemic has spread to this country. And, second, we employ a dummy that takes the value one in the week a country’s government issues lockdown policies in the form of stay-at-home orders as these put a stop to people’s economic activities. Once their value switches to one, both treatment variables remain unchanged in all following weeks. The key assumption underlying DiD is that of parallel trends, that is, treated and untreated units should not exhibit different trends before treatment (Angrist and Pischke, 2009), allowing us to attribute any post-treatment differences between treated and untreated units to the treatment. One way to evaluate this assumption is the inclusion of unit-specific time trends which, if parallel trends are indeed the case, should be jointly zero or at least not substantially alter the estimate of the treatment effect (Wing et al., 2018).

Here, this is only the case after conditioning on a set of control variables, namely, country-year-fixed effects $\alpha_{iy}$. These allow us to capture the effects of events and variables which are group-specific and time-variant but also relatively slow-moving, at least compared to the weekly time-structure of the panel we use. In this set-up, country-year-fixed effects also replace commonly used control variables measured at the country-year level such as economic development, regime type and population which are currently not available for 2019–2020. The DiD framework thus allows us to estimate the causal effects of Covid-19 and government responses to the pandemic on armed conflict while purging the confounding effects of all factors from the analysis that are either (1) non-country specific and highly time-varying over weeks or (2) country-specific but either time-invariant or relatively slow-moving, that is, varying over years. We use Poisson models to estimate the equation $\mathrm{battle}\;\;\mathrm{even}t_{iw}=\alpha_{iy}+\gamma_{w}+\beta T_{iw}+\varepsilon_{iw}$ where we are interested in the treatment effect β. We cluster standard errors on the country to account for serial correlation and overdispersion (Bertrand et al., 2004; Wooldridge, 1999).

## Results

**Figure 2.**
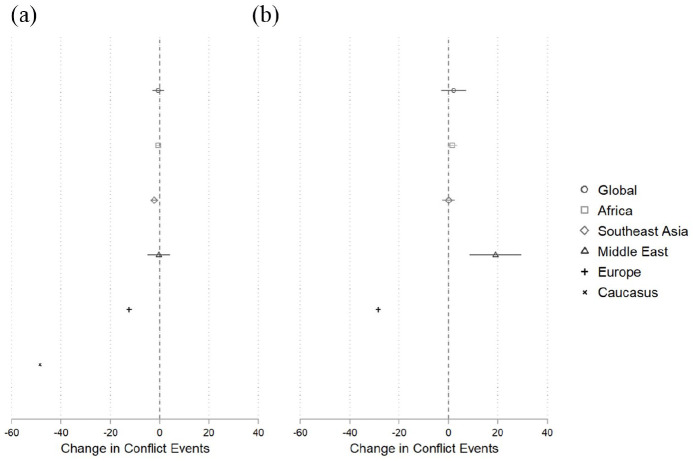
Covid-19 and Battle Events. (a) Treatment: First Case. (b) Treatment: Lockdown. First difference estimates, each estimate presents the treatment effect $\hat{\beta }$ from a separate model. Whiskers represent 95% confidence intervals.

The results of these models are shown in Figure 2.^3^ We present global as well as regional changes in the number of battles in the wake of (a) the first case of Covid-19 and (b) governmental stay-at-home orders.

Our results suggest that the spread of coronavirus has had no effect on global levels of armed conflict. For both treatment variables, the change in battle numbers is very close to zero, and the 95% confidence intervals, (–2.94, 1.90) in panel (a) and (–3.02, 7.23) in panel (b), include zero change. However, these global effects hide substantial regional variation. The results in panel (a) indicate that battle numbers decreased in Southeast Asia, Europe and the Caucasus in the wake of countries experiencing their first case of Covid-19. More worrisome, the results in panel (b) show that governmental lockdowns also decreased conflict in Europe *but increased fighting in the Middle East by an estimated 20 weekly battle events per country*.

In the Supplemental Appendix, we also present models that use battle fatality counts instead of battle event counts as dependent variable, which account for the potential effects Ramadan has had on fighting in majority Muslim countries, and that use Huber-White instead of clustered standard errors as the latter can be problematic if there are few clusters (Cameron et al., 2008). With the exception of both treatment effects becoming insignificant for Europe when Huber-White standard errors are used, our results remain substantively unchanged. While, at least in some countries, such as the Philippines, combatants thus initially heeded the UN Secretary-General’s call for restraint, our findings hence indicate that governmental lockdowns increase conflict in at least one volatile world region as countries such as Libya experience renewed fighting.

## Conclusion

In this study, we use real-time data on coronavirus and battle events to test the effect of the Covid-19 pandemic on global armed conflict. Initial descriptive analyses suggest a decrease in combat events, but our further analyses ultimately provide little evidence that Covid-19 has affected global armed conflict. However, we find heterogeneous effects across regions as battle numbers have decreased in some regions in the wake of the first reported cases while governmental lockdowns have increased conflict in the Middle East.

This finding is particularly concerning as we rely on *reported* battle numbers. As stressed by Metternich (2020) in the case of protests, it is likely that the pandemic has shifted attention away from armed conflict, is limiting journalists’ reporting ability as they self-distance, and has increased governmental capacities to suppress reporting on repression. This suggests that decreases in reported numbers are not due to an actual decrease in events but instead lowered reporting. As a result, we interpret the positive effect of government lockdowns on battles in the Middle East as conservative and caution against a too optimistic interpretation of the other, negative effects of the pandemic on armed conflict reported here.

Importantly, our study is limited to the short-term effects of Covid-19. As the social and economic repercussions of the pandemic will undoubtedly remain in the upcoming years, future studies should hence also examine its longer-term effects on armed conflict. In addition, future research should trace the source of the heterogeneous treatment effects we uncovered, that is, why did lockdowns result in conflict escalation in the Middle East but not in Southeast Asia?

In terms of policy recommendations, our research already indicates the importance of governmental measures seeking to curtail the spread of the pandemic being administered in tandem with measures that alleviate the economic impact these policies have on the vulnerable. Answering the open research questions outlined above will be crucial in stopping the potential vicious cycle involving the spread of the coronavirus pandemic and the intensification of armed conflict.

## Supplemental Material

Covid19_rn_appendixRR – Supplemental material for The Effect of the Covid-19 Pandemic on Global Armed Conflict: Early Evidence Supplemental material, Covid19_rn_appendixRR for The Effect of the Covid-19 Pandemic on Global Armed Conflict: Early Evidence by Marius Mehrl and Paul W Thurner in Political Studies Review

## Notes

1. We choose January 2018 as start date as Armed Conflict Location and Event Dataset (ACLED) did not track events in Central Asia and Europe before. Because of this, our analysis omits the Americas.

2. In contrast to the canonical difference-in-difference (DiD) equation, yiw = α + δTreati $+\gamma \mathrm{Pos}t_{w}+\beta \left(\mathrm{Trea}t_{i}\times \mathrm{Pos}t_{w}\right)+\varepsilon_{iw}$, this formulation does not distinguish between those units (countries in this application) which eventually receive treatment and those that do not. Instead, it only distinguishes between *observations* which have and have not received treatment as the time-invariant indicator for *units* which are treated at some point in time, $Treat_{i}$, and is subsumed in the country-fixed effects $\alpha_{i}$. Similarly, the indicator for such units having been treated, $Post_{w}$, is subsumed in the week-fixed effects $\gamma_{w}$. However, the coefficient of interest, β, which represents the effect of a unit receiving treatment remains unchanged. In the case of two units and two periods, the generalized DiD formulation reduces to the canonical one (Angrist and Pischke, 2009).

3. We present output tables for these models and alternative specifications in the Supplemental Appendix. In panel (b), the Caucasus-specific estimate is missing as there is no region-internal variation on the treatment variable.
