# Supplementary material for: The Effect of the Covid-19 Pandemic on Global Armed Conflict: Early Evidence
Source: Polit Stud Rev. 2021 May;19(2):286–93. doi: 10.1177/1478929920940648 (PMC7426721; doi:10.1177/1478929920940648)
Supplement: Covid19_rn_appendixRR – Supplemental material for The Effect of the Covid-19 Pandemic on Global Armed Conflict: Early Evidence [file Covid19_rn_appendixRR.pdf]

The Effect of the COVID-19 Pandemic on Global Armed Conflict: Early Evidence - Appendix

In this appendix, we present coefficient outputs for the models reported in the main analysis as well as alternative specifications. Table one presents results for the Poisson models using the first reported case of Covid-19 in a country as treatment variable while table two presents results for the Poisson models using the implementation of governmental stay-at-home policies as treatment variable. Table three replicates the main analysis using an alternative measure of conflict intensity, battle fatalities, as dependent variable instead of battle events. And figure one is used to inspect whether an outlier case drives the finding that fighting in the Middle East has intensified in the wake of governmental lockdowns.

The main models, as presented in the paper, are presented in columns three and nine where each coefficient represents the treatment effect as estimated in a separate model run on either the global sample or one of the regional subsamples. The models in columns one, two, seven, and eight present models where country-fixed effects are included instead of country-year-fixed effects. There, the inclusion of group-specific time trends substantially affects coefficient estimates in their significance and even direction, suggesting that the parallel trends assumption does not hold.

	(1)<br>DiD	(2)<br>DiD, group-specific time trends	(3)<br>DiD, country-year FE	(4)<br>DiD, country-year FE, group-specific time trend	(5)<br>DiD, country-year FE, Ramadan control	(6)<br>DiD, country-year FE, HW-standard errors
Global	-0.275	-0.179	-0.047	-0.052	-0.047	-0.047
	(0.159)	(0.133)	(0.114)	(0.110)	(0.114)	(0.138)
Africa	-0.256	-0.216	-0.233	-0.231	-0.233	-0.233
	(0.292)	(0.163)	(0.222)	(0.208)	(0.223)	(0.186)
Southeast Asia	-0.408	-0.079	-0.471*	-0.482*	-0.479*	-0.471*
	(0.209)	(0.183)	(0.219)	(0.218)	(0.214)	(0.219)
Middle East	-0.325	-0.366	-0.030	-0.046	-0.030	-0.030
	(0.222)	(0.213)	(0.215)	(0.212)	(0.215)	(0.318)
Europe	-1.039**	0.087**	-0.575**	-0.617**	-0.575**	-0.575
	(0.126)	(0.028)	(0.000)	(0.000)	(0.000)	(1.029)
Caucasus	-0.941**	-1.423**	-0.915**	-0.924**	-0.915**	-0.915**
	(0.021)	(0.059)	(0.000)	(0.006)	(0.000)	(0.058)
Country FE	yes	yes	no	no	no	no
Country-year FE	no	no	yes	yes	yes	yes
Week FE	yes	yes	yes	yes	yes	yes

Table one: Results from Poisson models, each estimate presents a coefficient from a separate model. Country-clustered standard errors in parentheses, ** p<0.01, * p<0.05

The models in columns four and ten then replicate those from columns three and nine while including group-specific time trends, here coefficient estimates stay substantively unchanged. Additionally, the models in columns five and eleven account for a potential effect of Ramadan, which in 2020 began during the Covid-19 pandemic, by including a dummy variable which takes the value one if an observation is during Ramadan and from a Muslim-majority country. Data on Muslim populations comes from the World Religion Data, we use the most recent 2010 numbers (Maoz and Henderson 2013). These results suggest that our results do not stem from capturing any potential effects of Ramadan as treatment coefficients remain substantively identical. Finally, the models in columns six and twelve use Huber-White (HW) standard errors as clustered ones can be problematic if there are few clusters (Cameron et al., 2008). Accordingly, these models indicate that the significant treatment effects for the region with the smallest number of clusters, Europe, are driven by using clustered Standard errors. Otherwise, the results in columns six and eleven remain unchanged from those reported in the main models in columns three and eight.

	(7)<br>DiD	(8)<br>DiD, group-specific time trends	(9)<br>DiD with country-year FE	(10)<br>DiD with country-year FE, group-specific time trend	(11)<br>DiD with country-year FE, Ramadan control	(12)<br>DiD, country-year FE, HW-standard errors
Global	0.164*	-0.029	0.148	0.076	0.155	0.148
	(0.073)	(0.087)	(0.171)	(0.163)	(0.170)	(0.107)
Africa	0.594	0.162	0.528	0.276	0.527	0.528*
	(0.312)	(0.263)	(0.272)	(0.289)	(0.270)	(0.209)
Southeast Asia	-0.081	0.213	-0.002	0.001	-0.010	-0.002
	(0.140)	(0.173)	(0.209)	(0.214)	(0.205)	(0.202)
Middle East	0.154	0.130	1.013**	0.946**	1.013**	1.013**
	(0.242)	(0.216)	(0.181)	(0.163)	(0.181)	(0.160)
Europe	-0.616**	-1.963**	-1.705**	-1.785**	-1.705**	-1.705
	(0.038)	(0.109)	(0.000)	(0.001)	(0.000)	(0.890)
Country FE	yes	yes	no	no	no	no
Country-year FE	no	no	yes	yes	yes	yes
Week FE	yes	yes	yes	yes	yes	yes

Table two: Results from Poisson models, each estimate presents a coefficient from a separate model. Caucasus-specific estimates are missing as there is no region-internal variation on the treatment variable. Country-clustered/HW standard errors in parentheses, ** p<0.01, * p<0.05

Our main models use the number of battle events in a country-week to measure the dependent variable conflict intensity. However, this is only one possible way to operationalize fighting intensity as other recent studies (e.g. Mehrl and Thurner 2020) use the number of battle fatalities instead. Instead, we rely on the number of battle events for the main analysis as data on this measure

of conflict intensity may be easier to collect than data on conflict casualties and thus less affected by the data quality problems discussed in the conclusion. Here, we nonetheless replicate our main models using the weekly number of combat fatalities, again taken from ACLED (Raleigh et al. 2010), as dependent variable; the models stay otherwise unchanged. Results for these models are presented in table three and substantively mirror those obtained in the main analysis.

	(13)<br>DiD with country-year FE<br>Treatment: First Case	(14)<br>DiD with country-year FE<br>Treatment: Lockdown
Global	-0.195	0.413
	(0.125)	(0.288)
Africa	-0.310	0.532*
	(0.167)	(0.224)
Southeast Asia	-0.066	-0.180
	(0.156)	(0.449)
Middle East	0.306	1.094**
	(0.393)	(0.234)
Europe	-1.578**	-2.197**
	(0.000)	(0.000)
Country FE	no	No
Country-year FE	yes	Yes
Week FE	yes	Yes

Table three: Results from Poisson models, each estimate presents a coefficient from a separate model. Dependent variable: Battle fatalities. Caucasus-specific estimates are missing as there is no region-internal variation on the treatment variable. Country-clustered standard errors in parentheses, ** p<0.01, * p<0.05

Finally, we inspect the battle event numbers in the Middle East underlying our finding that conflict there has increased in the wake of pandemic-induced governmental lockdowns to ensure that this result is not driven by outlier cases. To do so, we graph changes in Middle-Eastern countries average number of weekly battle events in the left panel of figure one and present boxplots of weekly battle numbers before and after lockdowns in its right panel. There, it becomes evident that three countries, Turkey, Iraq, and Syria, exhibit particularly clear differences in their pre- and post-lockdown fighting numbers. Whereas these differences are moderately sized and positive in Turkey and Iraq, i.e. 3.075 and 1.545, respectively, Syria emerges as a clear but *negative* outlier. That is, its average number of weekly battle events is 37.312 lower in the period after the government imposed a lockdown in mid-April than in the weeks before. This result is unsurprising given that some of the main conflict parties agreed to a ceasefire in Idlib in early March which subsequently resulted in a marked reduction of fighting (Rustad et al. 2020) but also suggests that our results on the Middle East are not driven by positive outlier observations. In line with this, the boxplots in the right panel of figure one also indicate that, if anything, outlier observations occurred before

lockdowns were instituted. The median number of weekly battle events varies little between the pre- and post-lockdown periods. However, the former period exhibits a wider range of values as some outlier country-weeks experienced up to 130 battles.

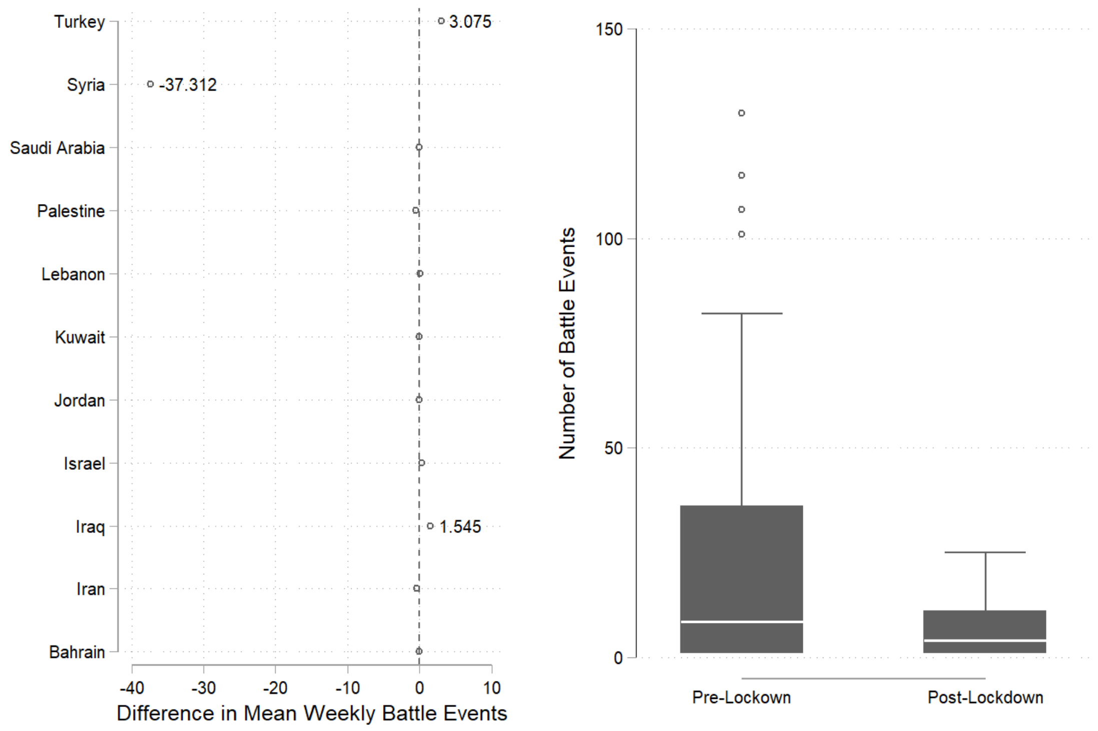

Figure one: The number of weekly battle events in in Middle-Eastern countries, pre- and post-lockdown. Pre-lockdown period includes observations from the year 2020 only. The right panel omits zero-observations for presentational purposes.

References

Cameron AC, Gelbach JB and Miller DL (2008) Bootstrap-Based Improvements for Inference with Clustered Errors. The Review of Economics and Statistics 90(3): 414–427.

Maoz Z and Henderson EA (2013) The World Religion Dataset, 1945-2010: Logic, Estimates, and Trends. International Interactions 39: 265-291.

Mehrl M and Thurner PW (2020) Military Technology and Human Loss in Intrastate Conflict: The Conditional Impact of Arms Imports. Journal of Conflict Resolution 64(6): 1172–1196.

Raleigh C, Linke A, Hegre H, et al. (2010) Introducing ACLED: An Armed Conflict Location and Event Dataset: Special Data Feature. Journal of Peace Research 47(5): 651–660.

Rustad SA, Nygård HM and Methi F (2020) *Are the Coronavirus Ceasefires Working?* PRIO Conflict Trends 04|2020. Oslo: Peace Research Institute Oslo. Available at: https://www.prio.org/utility/DownloadFile.ashx?id=2056&type=publicationfile (accessed 28 April 2020).
